# COVID-19 Vaccination Acceptance and Its Associated Factors Among a Middle Eastern Population

**DOI:** 10.3389/fpubh.2021.632914

**Published:** 2021-02-10

**Authors:** Walid A. Al-Qerem, Anan S. Jarab

**Affiliations:** ^1^Department of Pharmacy, Al-Zaytoonah University of Jordan, Amman, Jordan; ^2^Department of Clinical Pharmacy, Faculty of Pharmacy, Jordan University of Science and Technology, Irbid, Jordan

**Keywords:** knowladge, attitude, practice, COVID-19, vaccine

## Abstract

**Background:** The Coronavirus disease 2019 (COVID-19) pandemic is a major threat to public health and has had a significant impact on all aspects of life. An effective vaccine is the most anticipated resolution. This study aims to evaluate Jordanian intent to be vaccinated.

**Methods:** This is a cross-sectional web-based study. Sample characteristics were gathered, and the participants were classified according to the degree of COVID-19 risk based on the categories of the Centers for Disease Control and Prevention (CDC). Participants' KAP toward COVID-19 were assessed, and two scores were calculated: knowledge score and practice score. The association between different sample characteristics and these scores was identified using binary logistical regressions. The participants' vaccination intention was evaluated and multinomial logistic regression was applied to identify the predictors of vaccination intention. Finally, the reasons behind the participants' vaccination refusal/hesitation were determined and categorized into different groups.

**Results:** 1,144 participants were enrolled in the study (females = 66.5%). 30.4% of the participants were at high risk of COVID-19 complications, and 27.5% were at medium risk. Overall, participants' knowledge of COVID-19 symptoms, transmission methods, protective measures, and availability of cure were high (median of knowledge score = 17 out of 21). High protective practices were followed by many participants (median of practice score = 7 out of 10). 3.7% of participants were infected, and 6.4% suspected they were infected with the COVID-19 virus. 36.8% of the participants answered “No” when asked if they would take the vaccine once it becomes available, and 26.4% answered, “Not sure.” The main reasons for the participants' vaccination refusal or hesitancy were concerns regarding the use of vaccines and a lack of trust in them.

**Conclusion:** Participants reported high refusal/hesitancy. Several barriers were identified, and efforts should be intensified to overcome these barriers.

## Introduction

The Coronavirus disease 2019 (COVID-19) pandemic has been a health issue of great concern since 2020. Confirmed cases of the disease reached more than 35 million by October 2020 and have caused more than one million mortalities (1), particularly among the higher-risk population, including people who are obese, smokers, and patients that have cancer, chronic kidney disease, heart conditions, immunocompromised state, sickle cell disease, and type 2 diabetes mellitus (1).

In addition to the health impact of COVID-19, it has a significant economic burden that cannot be underestimated (2). It has caused a substantial reduction in workforces and an increase in unemployment globally (2). These negative impacts have encouraged pharmaceutical companies to develop a vaccine urgently. In December 2020, several vaccines were authorized to prevent COVID-19 infection (3), and more than 50 COVID-19 vaccine candidates were being developed (4). Vaccination has now begun in several countries around the world (5), with plans to begin vaccination in Jordan from February 2021 (6). Nevertheless, people still have doubts about the safety and efficacy of vaccines, including the longevity of protection against COVID-19, as several cases of reinfection have been reported (7, 8). Moreover, the rapid development of vaccines casts doubt on safety. Previously, the rapid development of vaccines has been linked to adverse issues. For example, the swine flu vaccine increased the risk of Guillain-Barré syndrome (9).

Vaccines have been a successful measure of disease prevention for decades (10). However, vaccine hesitancy and refusal are significant concerns globally, prompting the World Health Organization (WHO) to declare this uncertainty among the top 10 health threats in 2019 (11). The causes of vaccine hesitancy, as reported in different studies, include religious reasons, personal beliefs, and safety concerns due to wide-spread myths, including the association of vaccines and autism, brain damage, and other conditions (12). Unfortunately, in Jordan, no sufficient studies have been conducted to assess the Jordanian population's attitudes toward vaccination. To the best of our knowledge, no previously published work has evaluated the intent of Jordanians to be vaccinated against COVID-19 when a vaccine does become available. The present study aims to evaluate the intent of people from Jordan to be vaccinated against COVID-19 and evaluate the different sample characteristics associated with vaccine refusal/hesitancy, including KAP.

## Materials and Methods

This is a cross-sectional web-based survey. The enrolled participants were Jordanian in nationality and aged 18 years and above. A questionnaire was made using Google forms. The link was then distributed via different Jordanian all-purpose Facebook groups that included thousands of members. To ensure that participants met the inclusion criteria, questions about age, area of residence, and nationality were included in the questionnaire. Ethical approval was obtained from the Al-Zaytoonah ethical committee.

### Study Instrument

The questionnaire was developed based on a literature review. A panel of experts confirmed the content validity of the questionnaire. The questionnaire was developed in the English language and then translated to Arabic, which is the only official language in Jordan (98% of the Jordanian population are Arabs and the remaining 2% use Arabic for their daily interactions) (13). It was then translated back into English by different translators, and finally, compared by a third translator. Face validity was conducted in a pilot study that included 30 participants who assessed the questionnaire's clarity, and no significant modifications were required.

The final Arabic version of the questionnaire consisted of six branched sections. The first section collected participants' demographic information, including marital status, smoking habits, obesity status, education level, household average monthly income, health status, and whether participants worked in or studied a health-related field. The second section gathered information about the participants' experience with COVID-19. The third section assessed the attitudes of participants toward COVID-19, while the fourth evaluated their knowledge about COVID-19, including symptoms, transmission methods, preventive measures, and treatment availability. The fifth section asked about the preventive measures against COVID-19 used by the participants. The sixth and final section asked about the participants' willingness to be vaccinated against COVID-19 (once available), and the participants who responded “No” or “Not sure” were asked to give their reasons.

The degree of Covid-19 risk affecting participants was determined according to the Centers for Disease Control and Prevention (CDC) (1) categories. The high-risk group included smokers, obese, pregnant, or who had at least one of the following conditions (Type 2 diabetes mellitus/Chronic Obstructive Pulmonary Disease (COPD)/Cancer/Kidney Failure/Heart diseases/Organ transplantation/Sickle Cell Anemia). The medium-risk group included those who did not fit for the high-risk category but were overweight or had at least one of these conditions (Type 1 diabetes mellitus/Hypertension/Bone marrow transplant/ Cerebrovascular diseases or stroke/ Cystic Fibrosis/Asthma/Taking steroids or immunosuppressant drugs/Hepatic diseases/ Thalassemia/Lung fibrosis). The low-risk group included all other participants that do not fit the previously mentioned criteria.

Two scores were calculated: the knowledge score and the practice score. For knowledge, the maximum possible score was 21, as for each right answer, one point was granted (the score was calculated based on items in **Table 3**). The participants were divided into two groups based on their knowledge scores. The high-knowledge group included participants that scored more than the total sample median (median = 17), and the low-knowledge group included participants that scored below the total sample median.

Practice scores were calculated for those who had not been infected with the COVID-19 virus. One point was added for each answer representing a scientifically proven protective measure against COVID-19, and the maximum possible score was 10. After calculating the practice scores, the participants were divided into two groups. The high protective practice group included those who scored more than the sample median (median = 7), and the low protective practice group included those who scored below the median.

### Statistical Analysis

Kish formula (14) was applied to determine the least required sample size with a confidence interval level of 95% and a 4% margin for error. The estimated sample size was 600. Categorical variables were presented, such as frequency and percentages, and continuous variables were presented as means and standard deviations (SD). Crosstabulation with χ2 was applied to evaluate the association between intent to be vaccinated and participants' demographic characteristics, knowledge level about COVID-19, and protective practice against COVID-19. Binary logistic regressions were conducted on participants who answered “No” to “Have you ever been infected with COVID-19?” to evaluate variables associated with knowledge level and protective practice against COVID-19 level. A multinomial logistic regression was used to evaluate the variables related to the participants' intent to be vaccinated when a vaccine against COVID-19 becomes available.

An analysis of variance (ANOVA) with LSD *post hoc* test was applied to measure the difference in the perceived degree of seriousness of COVID-19 between participants with different responses to “Have you ever been infected with COVID-19?” All statistical analysis was conducted using SPSS version 25.

## Results

Sample characteristics are presented in Table 1. Thousand one hundred fourty four participants were enrolled in the study. Almost half of the participants (54.6%) were between 18–29 years, and 26.9% were between 30–40 years. 66.5% of the participants were female. Half of the participants were currently married, and 47.4% did not have children. Those with bachelor's degrees represented 53.5% of the sample, and <7% had a high school education or less. Most participants (67.2%) lived in Amman, and <4% lived in the southern governorates.

**Table 1:** Sample characteristics.

**Characteristic**		**Frequency (%)** **(*n* = 1,144)**
Age group	18–29 years	625 (54.6)
	30–40 years	308 (26.9)
	40–50 years	104 (9.1)
	50–60 years	55 (4.8)
	More than 60 years	52 (4.5)
Sex	Female	761 (66.5)
	Male	383 (33.5)
Marital status	Not married	558 (48.8)
	Married	586 (51.2)
Are you pregnant?	No	999 (98.9)
	Yes	11 (1.1)
Do you have children?	No	602 (52.6)
	Yes	542 (47.4)
Are you a smoker?	No	816 (71.3)
	Ex-smoker	52 (4.5)
	Yes	276 (24.1)
Weight status	Underweight	161 (14.1)
	Normal weight	533 (46.6)
	Overweight	392 (34.3)
	Obese	58 (5.1)
Residency	Amman	769 (67.2)
	Al Zarqa	179 (15.6)
	Southern governorates	39 (3.4)
	Northern governorates	157 (13.7)
Education level	High school or less	79 (6.9)
	Diploma	80 (7.0)
	University student	122 (10.7)
	Bachelor's degree	612 (53.5)
	Postgrad	251 (21.9)
Household average monthly income	<500	301 (26.3)
	500–1000	471 (41.2)
	More than 1000	372 (32.5)
Are you working/studying in a medical field?	No	605 (52.9)
	Yes	539 (47.1)

The participants' health status is outlined in Table 2. Fifteen percent of the participants had chronic diseases. Approximately one-third (30.4%) of the participants were at high risk of COVID-19 complications, and 27.5% were at medium risk.

**Table 2:** Participants' health status.

**Characteristic**	**Frequency(%)**
**Do you have any chronic diseases?**	
No	969 (84.7)
Yes	175 (15.3)
**Do you have any of the following diseases (Type 2 diabetes mellitus/Chronic Obstructive Pulmonary Disease (COPD)/Cancer/Kidney Failure/Heart diseases/Organ transplantation/Sickle Cell Anemia)?**	
No	130 (74.3)
Yes	45 (25.7)
**Do you have any of the following diseases? (Type 1 diabetes mellitus/Hypertension/Bone marrow transplant/ Cerebrovascular diseases or stroke/ Cystic Fibrosis/Asthma/Taking steroids or immunosuppressant drugs/Hepatic diseases/ Thalassemia/Lung fibrosis)**	
No	88 (50.3)
Yes	87 (49.7)
**Risk degree**	
High risk	348 (30.4)
Medium risk	315 (27.5)
Low risk	481 (42.0)

Table 3 represents the participants' knowledge about COVID-19 and vaccination. The most known symptom of COVID-19 was fever (97.6%), followed by the loss of smell and taste (96.8%), and the least known symptom was chills (70.1%). 99.2% of the participants were aware that the COVID-19 virus could be transmitted via the inhalation of respiratory droplets from an infected person. The most commonly known protective procedure amongst participants was social distancing (97.6%), followed by avoiding touching face/mouth/nose/eyes (95.4%) and using detergents (94.8%). 30.9% of the participants knew that zinc consumption could not prevent COVID-19 infection, and 15.6% knew the same fact about vitamin C.

**Table 3:** Participants' knowledge about COVID-19 and vaccination.

**Knowledge determents**	**Frequency(%)of** **choosing “Yes”**
**What are the symptoms of COVID-19?**	
Fever^**†**^	1,116 (97.6)
Chills^**†**^	802 (70.1)
Diarrhea^**†**^	815 (71.2)
Cough^**†**^	1,033 (90.3)
Otitis media^**‡**^	234 (20.5)
Loss of smell and taste senses^**†**^	1,107 (96.8)
No symptoms^**†**^	1,017 (88.9)
**How is COVID-19 transmitted?**	
Drinking unclean water^**‡**^	153 (13.4)
Eating unclean food^**‡**^	217 (19.0)
Inhalation of respiratory droplets of infected person^**†**^	1,135 (99.2)
Eating or touching wild animals^**‡**^	255 (22.3)
**What procedure do you think that may prevent COVID-19 infection?**	
Wearing face masks^**†**^	1,078 (94.2)
Washing hands with regular soup^**†**^	1,075 (94.0)
Using detergents^**†**^	1,084 (94.8)
Social distancing^**†**^	1,116 (97.6)
Avoid touching face/mouth/nose/eyes^**†**^	1,091 (95.4)
Consume vitamin C^**‡**^	966 (84.4)
Consume Zinc^**‡**^	791 (69.1)
Avoid eating meat^**‡**^	143 (12.5)
Consume herbs^**‡**^	594 (52.2)
**Believe that there is a cure for COVID-19**^**‡**^	22 (1.9)

† *Correct information*,

‡ *Incorrect information*.

Participants' attitudes and practices toward COVID-19 are shown in Table 4. Only 12.1% of the participants reported receiving the influenza vaccine last year. A fifth of the participants had done the PCR test to check if they had COVID-19, and 3.7% of the participants tested positive. 58.9% expect that they will be infected with COVID-19 but that their symptoms would be mild. In their response to their intention to take the COVID-19 vaccine once it was available, only 36.8% of the participants intend to be vaccinated, and 26.4% were not sure.

**Table 4:** Participants' attitudes and practices toward COVID-19 and vaccination.

**Variable**	**Frequency(%)**
**Did you take the influenza vaccine last year?**	
No	1,006 (87.9)
Yes	138 (12.1)
**Do you know someone close to you who has been infected with COVID-19?**	
No	534 (46.7)
Maybe	62 (5.4)
Yes	548 (47.9)
**Have you ever been infected with COVID-19?**	
No	1,014 (88.6)
Maybe	88 (7.7)
Yes	42 (3.7)
**In the event that you have COVID-19, are you ready to tell your relatives and friends?**	
No	52 (5.1)
Maybe	65 (6.4)
Yes	897 (88.5)
**In your opinion, what is the likelihood that you will be infected with Coronavirus during the next 6 months**	
I think I will be infected and my symptoms will be severe	73 (7.2)
I think I will be infected and my symptoms will be mild	597 (58.9)
I do not think that I will be infected	344 (33.9)
**If a vaccine is available for COVID-19, are you willing to take it?**	
No	421 (36.8)
Not sure	302 (26.4)
Yes	421 (36.8)
**What procedures have you done to protect yourself from COVID-19?**	
Wearing face masks^*^	1,101 (96.2)
Washing hands with regular soup^*^	1,091 (95.4)
Using detergents^*^	1,070 (93.5)
Social distancing^*^	1,035 (90.5)
Avoid touching face/mouth/nose/eyes^*^	936 (81.8)
Consume vitamin C^**^ (specifically as a protective measure)	727 (63.5)
Consume Zinc^**^ (specifically as a protective measure)	412 (36.0)
Avoid eating meat^**^ (specifically as a protective measure)	107 (9.4)
Consume herbs^**^ (specifically as a protective measure)	453 (39.6)
Have you tested to see if you have COVID 19 (PCR test)?^*^	256 (22.4)

* *Scientifically proven protective measure against COVID-19*,

** *Non-scientifically proven protective measure against COVID-19*.

The results of binary logistical regression between knowledge score and different sample characteristics are shown in Table 5. Not knowing someone infected with COVID-19 significantly decreased the odds of having high knowledge scores compared to those who knew someone infected (*p*-value = 0.01). A low or medium household monthly average income also considerably reduced the odds of having a high knowledge score compared to high income. Lastly, those with a high-risk of COVID-19 had significantly lower odds of getting high knowledge scores compared to those with low-risk degree.

**Table 5:** Binary logistical regression analysis of knowledge score.

	**B**	**S.E**.	**Wald**	**df**	***P***	**OR**	**95% CI for OR**	
							**Lower**	**Upper**
**Sex**								
Female	0.22	0.15	2.07	1.00	0.15	1.25	0.92	1.69
Male	Reference							
**Marital status**								
Not married	−0.28	0.24	1.44	1.00	0.23	0.75	0.47	1.20
Married	Reference							
**Age group**								
30–40 years	−0.06	0.17	0.11	1.00	0.75	0.95	0.68	1.32
40–50 years	0.08	0.26	0.10	1.00	0.76	1.08	0.65	1.79
50–60 years	−0.09	0.35	0.06	1.00	0.80	0.92	0.47	1.81
More than 60 years	0.07	0.33	0.05	1.00	0.82	1.08	0.57	2.05
18–29 years	Reference							
**Do you have children?**								
No	−0.05	0.24	0.04	1.00	0.85	0.96	0.60	1.52
Yes	Reference							
**Education level**								
High school or less	−0.37	0.30	1.45	1.00	0.23	0.69	0.38	1.26
Diploma	0.26	0.32	0.62	1.00	0.43	1.29	0.68	2.43
University student	−0.32	0.27	1.41	1.00	0.24	0.73	0.43	1.23
Bachelor's degree	0.26	0.19	1.97	1.00	0.16	1.30	0.90	1.87
Postgrad	Reference							
**Are you working/studying in a medical filed?**								
No	−0.28	0.15	3.65	1.00	0.06	0.75	0.56	1.01
Yes	Reference							
**Residency**								
Al Zarqa	−0.15	0.19	0.62	1.00	0.43	0.86	0.59	1.25
South governorates	−0.61	0.36	2.90	1.00	0.09	0.54	0.27	1.10
North governorates	0.13	0.20	0.43	1.00	0.51	1.14	0.77	1.70
Amman	Reference							
**Do you know someone close to you was infected with COVID-19?**								
No	−0.38	0.14	7.08	1.00	0.01^*^	0.69	0.52	0.91
Maybe	−0.79	0.31	6.57	1.00	0.01^*^	0.46	0.25	0.83
Yes	Reference							
**Risk degree**								
High risk	−0.37	0.17	4.39	1.00	0.04^*^	0.69	0.49	0.98
Medium risk	0.09	0.17	0.26	1.00	0.61	1.09	0.78	1.52
Low risk	Reference							
**Household average monthly income**								
Less than 500	−0.54	0.19	8.54	1.00	0.00^**^	0.58	0.40	0.84
500-1000	−0.45	0.16	7.46	1.00	0.01^*^	0.64	0.46	0.88
More than 1000	Reference							

*B, B coefficient; SE, standard error; Wald, Wald chi-square test; df, degrees of freedom; p, p-value; OR, odds ratio; CI, confidence interval*.

* *Significance taken at p < 0.05 (in BOLD)*,

** *Significance taken at p < 0.01 (in BOLD)*.

The results of binary logistical regression between practice score and different sample characteristics are shown in Table 6. Significant predictors of high protective practices were older groups (those who were older than 60 years compared to those between 18–29 years), not having children, residency (AlZarqa residents when compared to Amman residents), and higher knowledge score (*p*-values < 0.05). Meanwhile, the only significant predictor of low protective practices was being unmarried (*p*-value = 0.03).

**Table 6:** Binary logistical regression analysis of practice score.

	**B**	**S.E**.	**Wald**	**Df**	***p***	**OR**	**95% CI for OR**	
							**Lower**	**Upper**
**Sex**								
Female	0.01	0.16	0.00	1.00	0.97	1.01	0.74	1.38
Male	Reference							
**Age group**								
30-40 years	−0.22	0.16	1.89	1.00	0.17	0.80	0.58	1.10
40–50 years	−0.29	0.26	1.26	1.00	0.26	0.75	0.45	1.24
50–60 years	0.03	0.36	0.01	1.00	0.92	1.03	0.51	2.09
More than 60 years	0.88	0.35	6.41	1.00	0.01^*^	2.42	1.22	4.79
18–29 years	Reference							
**Marital status**								
Not married	−0.55	0.25	4.74	1.00	0.03^*^	0.58	0.35	0.95
Married	Reference							
**Do you have children?**								
No	0.55	0.25	4.66	1.00	0.03^*^	1.73	1.05	2.83
Yes	Reference							
**Are you working/studying in a medical filed?**								
No	−0.08	0.15	0.29	1.00	0.59	0.92	0.68	1.24
Yes	Reference							
**Residency**								
Al Zarqa	0.39	0.20	4.01	1.00	0.045^*^	1.48	1.01	2.18
South governorates	−0.30	0.39	0.61	1.00	0.43	0.74	0.34	1.58
North governorates	0.27	0.21	1.60	1.00	0.21	1.30	0.86	1.96
Amman	Reference							
**Do you know someone close to you was infected with COVID-19?**								
Maybe	0.17	0.32	0.29	1.00	0.59	1.19	0.63	2.22
No	−0.08	0.14	0.29	1.00	0.59	0.93	0.70	1.23
Yes	Reference							
**Risk degree**								
High risk	0.24	0.18	1.68	1.00	0.20	1.27	0.89	1.81
Medium risk	−0.25	0.17	2.15	1.00	0.14	0.77	0.55	1.09
Low risk	Reference							
**Household average monthly income**								
<500	−0.15	0.19	0.66	1.00	0.42	0.86	0.60	1.24
500–1000	−0.09	0.16	0.30	1.00	0.58	0.91	0.66	1.26
More than 1000	Reference							
**Knowledge score**	0.40	0.04	99.84	1.00	<0.001^**^	1.50	1.38	1.62
**In your opinion, what is the likelihood that you will be infected with corona virus during the next 6 months**								
I think I will be infected and my symptoms will be severe	0.12	0.29	0.18	1.00	0.67	1.13	0.64	2.01
I think I will be infected and my symptoms will be mild	0.00	0.15	0.00	1.00	0.98	1.00	0.74	1.35
I do not think that I will be infected	Reference							
**Estimation of disease seriousness**	0.02	0.03	0.32	1.00	0.57	1.02	0.96	1.08

B, B coefficient; SE, standard error; Wald, Wald chi-square test; df, degrees of freedom; p, p-value; OR, odds ratio; CI, confidence interval.

* *Significance taken at p < 0.05 (in BOLD)*,

** *Significance taken at p < 0.01 (in BOLD)*.

The χ2 test assessed the association between the sample characteristics and the participant's intent to be vaccinated (Appendix) and revealed that the participants' characteristics were significantly associated with the response of “No” vs. “Yes,” when they were female, married, having children, and had a diploma degree. On the other hand, acquaintance with someone who was infected with COVID-19 was significantly associated with the response of “Yes” vs. “No” and “Yes” vs. “Not sure” (*p*-values 0.01 and 0.03, respectively). Higher percentages of those who work/study in the medical field responded “Yes” rather than “Not sure” (39.7 vs. 23.9%). Those who wore face masks and used detergents, but did not consume vitamin C to protect themselves from COVID-19, had a higher percentage of “Yes” vs. “No” (*p*-values= 0.002, 0.02, and 0.04, respectively). No significant difference was found in the vaccine acceptance between the participants with different risk degrees for COVID-19 complications (Percentage of responding “Yes” was 37.1 % in the high-risk group, 35.9% in the medium-risk group, and 37.2% in the low-risk group).

Table 7 shows the multivariate predictors of responding “Not Sure” or “No” regarding the intent to be vaccinated, according to the multinomial model. Female participants had a 3-fold higher relative likelihood of responding “No” vs. “Yes” and a nearly 1.5-fold higher relative chance of responding “Not sure” vs. “Yes” when compared with male participants (*p*-values < 0.05). Moreover, a significant association was found between the participants' perception of the seriousness of COVID-19 and his/her intention to be vaccinated. The higher the perceived seriousness of COVID 19, the significantly lower the odds of responding “Not sure” or “No.”

**Table 7:** Multivariate predictors of responding “not sure” or “no” regarding intent to be vaccinated.

**Characteristic**	**Intent to be** **vaccinated:** **No vs. Yes**	**Intent to be** **vaccinated:** **Not sure vs. Yes**
	**OR (95% CI)**	**OR (95% CI)**
**Age**		
18–29 years	0.74(0.36-1.54)	1.1(0.48-2.51)
30–40 years	0.8(0.37-1.71)	1.23(0.53-2.88)
40–50 years	1.25(0.52-3.00)	0.81(0.29-2.28)
50–60 years	1.09(0.39-3.05)	1.14(0.36-3.60)
More than 60 years	Reference	
**Sex**		
Female	3.00^**^(2.07–4.36)	1.49^*^(1.03–2.16)
Male	Reference	
**Education level**		
High school or less	0.52(0.26–1.02)	0.7(0.34–1.46)
Diploma	0.69(0.34–1.41)	0.55(0.24–1.27)
University student	0.49^*^(0.26–0.91)	0.58(0.31–1.11)
Bachelor's degree	0.68(0.44–1.03)	0.87(0.56–1.36)
Postgrad	Reference	
**Marital status**		
Not married	0.45^**^(0.26–0.77)	0.65(0.36–1.15)
Married	Reference	
**Do you have children?**		
No	1.07(0.62–1.83)	1.06(0.59–1.90)
Yes	Reference	
**Risk degree**		
high risk	1.02(0.68–1.53)	0.95(0.62–1.46)
medium risk	0.89(0.60–1.31)	1.04(0.70–1.56)
low risk	Reference	
**In your opinion, what is the likelihood that you will be infected with coronavirus during the next 6 months**		
I think I will be infected and my symptoms will be severe	0.57(0.29–1.12)	1.06(0.56–1.97)
I think I will be infected and my symptoms will be mild	0.83(0.59–1.16)	0.87(0.61–1.24)
I do not think that I will be infected	Reference	
**In your opinion, how serious is COVID-19?**	0.75^**^(0.70–0.81)	0.88^**^(0.81–0.95)
**Total knowledge**	1.01(0.94–1.10)	1(0.92–1.08)

OR, odds ratio; CI, confidence interval.

* *Significance taken at p < 0.05 (in BOLD)*,

** *Significance taken at p < 0.01 (in BOLD)*.

Table 8 represents the attitudes and practices of those who were infected with COVID-19 or suspected infection. Those who were infected adhered to quarantine procedures significantly more than those who suspected they were infected. Moreover, confirmed cases of COVID-19 tended to tell relatives/friends more about infection, compared to those who suspected infection. ANOVA with *post hoc* analysis indicated that there were no significant differences in the perception of COVID-19 seriousness between the three groups (infected, suspected infection, and not infected with COVID-19).

**Table 8:** Attitudes and Practices of the participants who were infected or suspected their infection.

**Variable**	**Answered yes to ever had** **COVID-19**	**Answered maybe to ever had** **COVID-19**	***P*-value**
Degree of adherence with quarantine (0 not at all, 10 completely)	9.00(2.12)	7.61(2.77)	0.005^*^
Seriousness of COVID-19 (0 not at all, 10 extremely)	6.57(2.17)	6.52(2.28)	0.91
Degree of anxiety (0 not at all, 10 extremely)	5.93(2.90)	5.97(2.82)	0.94
**Did you tell your relatives/friends when suspected/knew that you had COVID-19?**			<0.001^*^
Yes	40(95.2)	58(65.9)	
No	2(4.8)	30(34.1)	

* *Significance taken at p < 0.01 (in bold)*.

The reasons participants' did not want to take the vaccine or were hesitant about vaccination are shown in Table 9. The most mentioned reasons were concerns about the vaccines as 98.3% of those who answered “No” and 99.3% of those who answered “Not sure” had at least one concern. Concerns about the efficacy of the vaccine and its newness were the most reported by the participant, while the least reported concern was about the association between vaccination and autism (7.8%). The second most mentioned reasons represented attitudes toward vaccines. 52.3% of those who answered “No” stated that they do not take vaccines at all. The need for additional information was a cause for answering “No” or “Not sure” for 87.9 and 97.4%, respectively, of the participants. Lack of trust was another reason for refusal or hesitation about taking the vaccine once available as 81% and 66.2%, respectively, of those who answered “No” or “Not sure” believed that the vaccine might have been approved too quickly because of political pressure.

**Table 9:** Reasons participants provided for responding “no” or “not sure” regarding intent to be vaccinated.

**Reasons**	**Total**	**Intent to be vaccinated**	
		**No**	**Not sure**
**Concerns about the vaccine**	**714 (62.4)**	**414 (98.3)**	**300 (99.3)**
I am concerned about the vaccine efficacy	608 (53.1)	359 (85.3)	249 (82.5)
I am concerned about the vaccine safety and side effects	679 (59.4)	400 (95)	279 (92.4)
It might transmit the virus to me	398 (34.8)	266 (63.2)	132 (43.7)
The vaccine will be new, I won't be the first to get the vaccine	629 (55)	356 (84.6)	273 (90.4)
I am concerned about the vaccine rigor of testing	613 (53.6)	372 (88.4)	241 (79.8)
The vaccine may contain heavy metals or odd materials	508 (44.4)	337 (80)	171 (56.6)
Vaccines cause autism	89 (7.8)	62 (14.7)	27 (8.9)
The vaccine may affect fertility	137 (12)	92 (21.9)	45 (14.9)
Not convinced that it will be effective, look at the flu vaccine	324 (28.3)	224 (53.2)	100 (33.1)
My immune system is weak and I can't take inactivated vaccines/I have an allergy to many substances and I may have an allergy to this vaccine	135 (11.8)	81 (19.2)	54 (17.9)
I don't think that I can afford the vaccine	194 (17)	101 (24)	93 (30.8)
**Need additional information**	**664 (58.0)**	**370 (87.9)**	**294 (97.4)**
It depends on what my doctor recommends	371 (32.4)	153 (36.3)	218 (72.2)
It depends on the scale of the pandemic at the time of the vaccine. If very low, I may not do it	472 (41.3)	249 (59.1)	223 (73.8)
I don't want a vaccine I know nothing about. I'll make my decision if/when one becomes available	623 (54.5)	356 (84.6)	267 (88.4)
**Attitudes**	**670 (58.6)**	**369 (94.1)**	**274 (90.7)**
I don't feel I'm at risk	178 (15.6)	128 (30.4)	50 (16.6)
I am religious and God will protect me	193 (16.9)	125 (29.7)	68 (22.5)
I don't take vaccines at all	331 (28.9)	220 (52.3)	111 (36.8)
I am scared to put foreign objects in my body	450 (39.3)	301 (71.5)	149 (49.3)
I would say that the vaccine should go to the people who are most risk of contracting it before I get it because I am not putting myself at risk	512 (44.8)	281 (66.7)	231 (76.5)
**Lack of trust**	**628 (54.9)**	**392 (93.1)**	**236 (78.1)**
Any vaccine made for this virus I do not trust	399 (34.9)	296 (70.3)	103 (34.1)
If the government recommended it use I will not take it	352 (30.8)	268 (63.7)	84 (27.8)
There is no way I trust big pharmaceutical companies	290 (25.3)	206 (48.9)	84 (27.8)
I'm thinking a vaccine now might be approved too quickly because of political pressure	541 (47.3)	341 (81)	200 (66.2)
I believe that this virus was developed by the governments and I won't take any vaccine	280 (24.5)	206 (48.9)	74 (24.5)
Because I heard the government was to put a chip in you when you get the vaccination and I do not want a chip inside of me	166 (14.5)	111 (26.4)	55 (18.2)
**Others**			
I am afraid of needles	140 (12.2)	85 (20.2)	55 (18.2)

*The bold values indicate the groups of reasons for vaccine hesitancy/refusal*.

## Discussion

In recent history, vaccination has played an essential role in reducing the burden of infectious diseases. It prevented 33,000 deaths and 14 million diseases in 2001 (15). Vaccines from different companies, including Pfizer BioNTech, Moderna, and Oxford AstraZeneca have recently been approved, but their distribution is still limited (16). Identifying the populations' intention to be vaccinated, and the barriers to vaccination could remove these barriers and increase the vaccination rate once the vaccine is widely available.

### Attitudes and Practices of Participants Infected With COVID-19 or Suspected That They Were

The participants' perception of the seriousness of COVID-19 among participants was not associated with COVID-19 infection history. The degree of seriousness estimations were not different between those who were not infected, those who suspected their infection, and those who were infected (means = 6.49, 6.52, and 6.57, respectively). High adherence to quarantine was reported by those infected, which contradicts the results of other studies (17, 18), which reported poor adherence to quarantine during different pandemics. However, the degree of quarantine adherence among those who were certain about their infection was significantly higher than those who only suspected that they were infected. This indicates the importance of COVID-19 testing in decreasing the disease spread by increasing the adherence of infected patients to quarantine.

### Vaccination Intentions

Even though this study was conducted in October 2020 when the number of COVID-19 cases increased rapidly in Jordan, only 36.8% of participants intend to be vaccinated once the vaccine was available. This percentage is much lower than the percentage reported by a global survey that included participants from 19 countries (19) (71.5%) and by studies conducted in Ecuador (20) (97%), the United States (21) (57%), France (22) (76%), China (23) (91.3%), and Saudi Arabia (24) (64.7%). The peak rise in COVID-19 cases happened much earlier in other countries than in Jordan, which may contribute to the higher vaccination hesitancy among the Jordanian population compared to other populations, as the disease is new to Jordan. Although the percentage of the population who need to be vaccinated to achieve herd immunity against COVID-19 is not yet well-known, in general, 50–90% (25) of the population needs to be immune either naturally or by vaccines to achieve herd immunity. Should this high hesitancy toward vaccination continue among the Jordanian population, it might be difficult to achieve herd immunity. Several sample characteristics had a significant negative impact on the participants' intention to be vaccinated, including; being female, married, and having a postgrad degree compared to university students. Lower vaccination intention among female participants was also observed in studies conducted in France (22), China (23), and Europe (26). However, other factors reported in other studies, like income (22, 26) and age (22), were not significant predictors in this study. The recognition of these factors could help develop targeted awareness campaigns directed to the population to increase the vaccination rate once the vaccine is available.

The perceived risk of COVID-19 was a significant predictor of the participants' vaccination intention in this study, reflecting several other studies (22, 23). The higher the perceived risk, the lower the vaccination hesitancy. Therefore, increasing the population's consciousness about the seriousness of the disease is essential in improving their willingness to be vaccinated.

### Barriers

Detecting the causes of vaccination refusal or hesitancy could improve the population's vaccination intentions. It is important to better understand the rationales and reasons for vaccination refusal or hesitancy if we are to remove these barriers.

Concerns about the vaccine were the most common reason behind hesitancy or refusal among the participants. These concerns about vaccine safety and side effects are global, as indicated by studies conducted in the United States (21), Europe (26), and China (23). The rationale behind these concerns is reasonable, as several vaccine candidate trials were paused (27, 28) due to detected side effects. However, the suspension of these studies, once side effects were noted, could be used to assure the rigor of vaccine testing, another concern among the population. Misbeliefs about the association between vaccines and autism or the vaccine's effect on fertility were not common among the participants, implying the dubiety about many popular myths associated with vaccines.

Efficacy is a frequently mentioned concern whenever a new vaccine is developed (29, 30). This concern could be of less importance once the vaccine is available and successful results are published.

Undesirable attitudes were the second most mentioned barriers. Increasing the population's understanding of the vaccines and the related mechanisms of action through different awareness-raising methods could overcome this barrier. About 30% of those with undesirable attitudes were against vaccination in general. Several studies (31, 32) have established approaches to overcome vaccine refusal that could be useful for COVID-19 vaccination. For example, opposing the spread of false information and targeting children and adolescence, who might not have robust emotions about vaccines yet, could increase COVID-19 vaccine acceptability.

The need for additional information was reported as a barrier by 58% of the respondents and 79% of participants in an Indonesian study (33). The role of healthcare providers is influential in this respect, as they provide patients and the general population with much-needed information. An Australian study (34) provided a framework that could be used by healthcare providers to increase confidence in any potential COVID-19 vaccines.

Lack of trust was also a cause of vaccination hesitation or refusal for many participants in this study and an American study (21). A belief in the conspiracy theories associated with COVID-19 among the Jordanian population was observed in the present study. These beliefs have also been reported by another Jordanian study on COVID-19 (unpublished data). Several strategies have been suggested to combat conspiracy theories (35), including the careful dissemination of medical research, social media campaigns, and developing a culture of fact-checking. A report issued by WHO has discussed the behavioral considerations of COVID-19 acceptance and suggested different approaches to increasing vaccine acceptance. These include building an enabling environment and using open communication to address people's beliefs and uncertainty, educating them about the safety and efficacy of the vaccine (36). Social and governmental collaboration will increase public confidence in the COVID-19 vaccine and enable the country to reach herd immunity rapidly.

### Strengths and Limitations

One of this study's strengths is the large sample size, which decreases the influence of existing bias. Another strong aspect of the present study is that it evaluated the participants' KAP toward COVID-19 and their vaccination intentions and assessed the association between KAP and vaccination intentions.

At the time this study was conducted, the vaccine was not available. The participants' vaccination intentions may be different now, as the vaccine has been made available. More information has now been published, which may be considered a limitation of this study. This study was based on an online questionnaire, meaning the results are subject to recall and selection biases. However, previous studies have shown that web-based research is a cost-effective method that can be used to generate a sample that is representative of the total population with a fraction of the cost (37). It can reach people otherwise unreachable and provides a safe and private environment for the respondents to answer questions accurately and honestly compared with face-to-face interviews (38).

It has been suggested that as the number of Internet users has increased globally, the socio-demographic characteristics of the recruited participants via web-based surveys reflect the general population (39). This can be applied in Jordan as Internet users are estimated to be 67% of all age groups (40). This percentage could be higher when children under the age of 18 are excluded. Another limitation could be that the study sample age was positively skewed. However, the Jordanian population is a young one, and the age group between 20–29 years represents 33.45% of the total population above the age of 19 (41).

Finally, almost half of the sample participants worked in the medical field, which may cast doubt in the sample's representation of the total population. Nevertheless, the percentage of medical field workers in Jordan is significantly higher than in other countries globally. For example, Jordan is ranked fourth in terms of the number of pharmacists for every 10,000 people (16 pharmacists for 10,000) (42). Furthermore, the authors believe that the perception of medical-related staff is particularly interesting, as their opinions influence the general population, and they represent a high-risk group, therefore, their vaccination is a priority. Finally, as indicated by the Kish formula, the smallest required sample size is 600, therefore, a study sample that includes 1,144 participants could provide sufficient data to evaluate each subgroup.

## Conclusion

The results of this study indicate that the study sample has good KAP toward COVID-19. However, the participants' vaccination intentions were unfavorable. The total sample acceptance of the vaccine was 36.8%, while the approval of the participants who work/study in a medical field or those who are at high risk of COVID-19 complications was slightly higher (39.7 and 37.1%, respectively). The main reasons for participants' refusal of vaccination or hesitation were concerns about safety and efficacy, in addition to insufficient information about the vaccine. Healthcare providers must activate their roles and address these concerns by increasing awareness about the role of vaccination in preventing the spread of infection and acquiring herd immunity. This could be achieved by designing and implanting different awareness campaigns via various media outlets guided by healthcare providers.

## Data Availability Statement

The raw data supporting the conclusions of this article will be made available by the authors, without undue reservation.

## Ethics Statement

The studies involving human participants were reviewed and approved by Al Zaytoonah University of Jordan ethical committee. The patients/participants provided their written informed consent to participate in this study.

## Author Contributions

WA-Q: methodology, formal analysis, data curation, original draft preparation, writing, review and editing, funding acquisition, supervision, and final approval of the submitted manuscript. AJ: draft preparation, writing, review and editing, and final approval of the submitted manuscript. All authors contributed to the article and approved the submitted version.

## Conflict of Interest

The authors declare that the research was conducted in the absence of any commercial or financial relationships that could be construed as a potential conflict of interest.
